# Plant height heterosis is quantitatively associated with expression levels of plastid ribosomal proteins

**DOI:** 10.1073/pnas.2109332118

**Published:** 2021-11-15

**Authors:** Devon Birdseye, Laura A. de Boer, Hua Bai, Peng Zhou, Zhouxin Shen, Eric A. Schmelz, Nathan M. Springer, Steven P. Briggs

**Affiliations:** ^a^Division of Biological Sciences, University of California San Diego, La Jolla, CA 92093;; ^b^Department of Plant and Microbial Biology, University of Minnesota, Saint Paul, MN 55108

**Keywords:** heterosis, hybrid vigor, proteomics, ethylene, maize

## Abstract

The use of hybrids is widespread in agriculture, yet the molecular basis for hybrid vigor (heterosis) remains obscure. To identify molecular components that may contribute to trait heterosis, we analyzed paired proteomic and transcriptomic data from seedling leaf and mature leaf blade tissues of maize hybrids and their inbred parents. Nuclear- and plastid-encoded subunits of complexes required for protein synthesis in the chloroplast and for the light reactions of photosynthesis were expressed above midparent and high-parent levels, respectively. Consistent with previous reports in *Arabidopsis*, ethylene biosynthetic enzymes were expressed below midparent levels in the hybrids, suggesting a conserved mechanism for heterosis between monocots and dicots. The ethylene biosynthesis mutant, *acs2/acs6*, largely phenocopied the hybrid proteome, indicating that a reduction in ethylene biosynthesis may mediate the differences between inbreds and their hybrids. To rank the relevance of expression differences to trait heterosis, we compared seedling leaf protein levels to the adult plant height of 15 hybrids. Hybrid/midparent expression ratios were most positively correlated with hybrid/midparent plant height ratios for the chloroplast ribosomal proteins. Our results show that increased expression of chloroplast ribosomal proteins in hybrid seedling leaves is mediated by reduced expression of ethylene biosynthetic enzymes and that the degree of their overexpression in seedlings can quantitatively predict adult trait heterosis.

## Significance

Heterosis (hybrid vigor) boosts the productivity and resilience of crops and livestock above the levels of both parents, yet its underlying mechanisms remain unknown. We analyzed expression patterns of proteins in maize hybrids and their inbred parents. Differences in several molecular machines and biochemical pathways were found and quantitatively assessed using a panel of 15 hybrids. Seedling leaf chloroplast ribosomal proteins were able to quantitatively infer levels of adult plant heterosis. Expression levels of biosynthetic enzymes for the stress hormone ethylene were reduced in hybrids, as was previously reported for the dicot *Arabidopsis*. Mutation of these genes in a maize inbred caused the proteome to resemble a hybrid. Repression of ethylene biosynthesis may be a conserved component of heterosis physiology.

Plants, animals, and humans display inbreeding depression associated with increased genetic uniformity and characterized by reduced vigor (1). Mating between genetically distinct inbred parents can produce hybrid vigor, or heterosis, defined as the difference in vigor between a hybrid and 1) the average of its parents or 2) the better-performing parent. Hybrids may outperform their parents in terms of size, vigor, yield, abiotic and biotic stress resistance, longevity, and reproductive advantage (2, 3). Maize is the most productive crop in the United States, and it was the first hybrid crop to be made and sold. Heterosis in maize increases many traits far above the levels of the higher parent, including biomass and harvestable grain (4). Despite its importance in agriculture, the changes in physiology that cause hybrid vigor remain obscure. Breeding hybrids requires expensive and labor-intensive field tests of both the hybrids and their parents. Progress in maize breeding has greatly improved yield through increased tolerance of high-density planting stress, and this primarily constitutes the nonheterosis portion of yield (5). It is unclear whether substantial improvements in heterosis are possible and yet have lagged because breeders lack seedling biomarkers for adult plant heterosis.

Quantitative trait locus mapping has shown that minor effect loci for heterosis are distributed throughout the genome (1). Transcriptome (6) and proteome (7) profiles indicate that gene expression levels in hybrids are generally the average of their parents. A minority of messenger RNAs (mRNAs) and proteins are expressed above or below midparent (MP) levels, and most of these gene products are not obviously related to each other in function or to hybrid phenotypes, making their unusual levels difficult to interpret (8). Despite these ambiguities, progress has been made in understanding hybrid vigor.

Recent work has implicated the plant hormone ethylene (ET) in heterosis. In *Arabidopsis* hybrids, ET is produced at reduced levels, and application of exogenous ET reduced hybrid vigor (9). Thus, ET potentially mediates at least some aspects of heterosis, though it is unclear which proteins are affected by reduced ET biosynthesis.

Maize hybrids have greater photosynthetic capacity than their inbred parents (10), which presumably contributes to their increased biomass and yield. In both maize and *Arabidopsis*, various genes for photosynthesis have been observed to be expressed above MP levels (10–12). Some metabolites and enzymes of the photosynthetic carbon reactions are elevated in maize hybrids, while others in photorespiration are repressed (13). We sought to identify protein biomarkers in seedling leaves that contribute insights to the physiology of hybrid vigor and can be used to predict levels of adult trait heterosis.

## Results

### Expression Patterns of Chloroplast Protein Complexes.

Multiplexed proteomic analyses were performed by using tandem mass tag (TMT) peptide tags and high-resolution mass spectrometry to quantify differences in the levels of proteins extracted from seedling leaves of hybrids and their inbred parents. To control for developmental differences between hybrids and inbreds, we utilized staggered planting dates to ensure that the plants were all at the same developmental stage on the same date of sampling. Expression levels in the hybrid, B73×Mo17, were compared to high-parent (HP) levels, low-parent (LP) levels, and calculated MP levels to identify nonadditive expression.

**Fig. 1.**
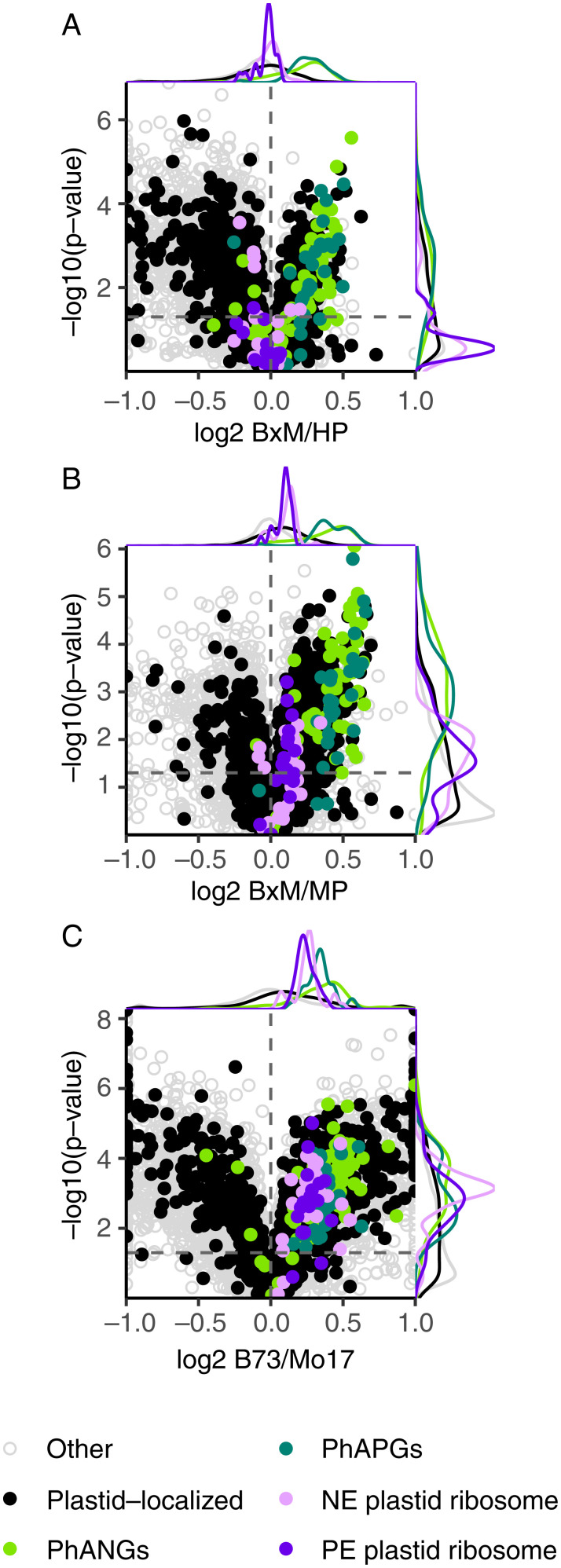
Volcano plots displaying expression patterns of the most significantly nonadditive proteins, representing B73×Mo17/HP (*A*), B73×Mo17/MP (*B*), and B73/Mo17 (*C*). Points to the left of the vertical dotted line correspond to below HP (*A*), below MP (*B*), or Mo17 HP (*C*) proteins; points to the right of the vertical dotted line correspond to above HP (*A*), above MP (*B*), or B73 HP (*C*) proteins. PhANGs, PhAPGs, nuclear-encoded (NE) plastid ribosome, and plastid-encoded (PE) plastid ribosome proteins are color-coded.

Of 10,141 measured leaf proteins, 638 (6%) were expressed above HP. Functional enrichment performed by using The Database for Annotation, Visualization and Integrated Discovery (14, 15) revealed that these were enriched for chloroplast localization. Of the 1,068 chloroplast-localized proteins observed, 256 (24%) were expressed above HP levels. This included 65 of the Photosynthesis-Associated Nuclear Genes (PhANGs) and Photosynthesis-Associated Plastid Genes (PhAPGs). The PhAPG/PhANG proteins comprise photosystem I, photosystem II (PSII), cytochrome b6/f complex, NAD(P)H dehydrogenase-like complex, adenosine triphosphate (ATP) synthase, and the carbon-fixation enzyme, Rubisco. PhANG and PhAPG proteins were expressed 19% above HP levels, on average (Fig. 1*A*). The remaining 189 chloroplast-localized proteins were weakly enriched for carbon fixation.

Expression between MP and HP levels was observed for 956 proteins (9%). They were enriched for plastid ribosomal proteins, encoded by either the nuclear or plastid genome. The ribosome subunits were expressed 8% above MP, on average (Fig. 1*B*). Less enrichment was observed for enzymes of carbon metabolism. While not enriched, carbon-fixation-related proteins were differentially expressed in the hybrid, some above MP and some below (*SI Appendix*, Fig. S1).

Nearly all plastid-encoded proteins were overexpressed in the hybrid, including translation initiation factor 1, chloroplast envelope membrane protein, the PhAPGs, and the ribosomal proteins (*SI Appendix*, Table S1). The remaining plastid-encoded proteins were expressed at MP levels, except for RNA polymerase alpha subunit, which was expressed below MP.

Proteins that were expressed nonadditively were frequently expressed at different levels between the parents, and expression in B73 was generally higher than in Mo17 (Fig. 1*C*). However, some proteins, such as photosynthetic electron transfer C, ribose-5-phosphate isomerase, and the oxygen-evolving complex assembly proteins, were expressed higher in Mo17 (Dataset S1). Statistical analyses for the differential expression of protein groups shown in Fig. 1 are provided in Dataset S2.

Expression below LP levels was observed for 517 proteins (5%). They were weakly enriched for amino acid biosynthesis, fatty acid degradation, chaperone proteins, and peroxisomal proteins. Expression between MP and LP was observed for 1,222 proteins (12%). These showed weak enrichment for chaperone proteins and protein processing in the endoplasmic reticulum.

The nonadditive patterns of expression seen in the proteome of juvenile leaves were also observed in the blades of mature leaves (*SI Appendix*, Fig. S2*A*). However, there was a reversal of relative expression levels between the parents for plastid ribosomal proteins (*SI Appendix*, Fig. S2*B*). B73 was HP in the seedling leaf, whereas Mo17 was HP in the leaf blade. Comparison of the total proteomes between the seedling leaf and the adult leaf blade revealed large differences associated with growth and development (*SI Appendix*, Fig. S3). Nevertheless, the proteome differences between the inbred parents and their hybrid were nearly identical at both stages of growth (*SI Appendix*, Fig. S2*A*).

### Correlations between Trait Heterosis and Protein Expression Heterosis.

**Fig. 2.**
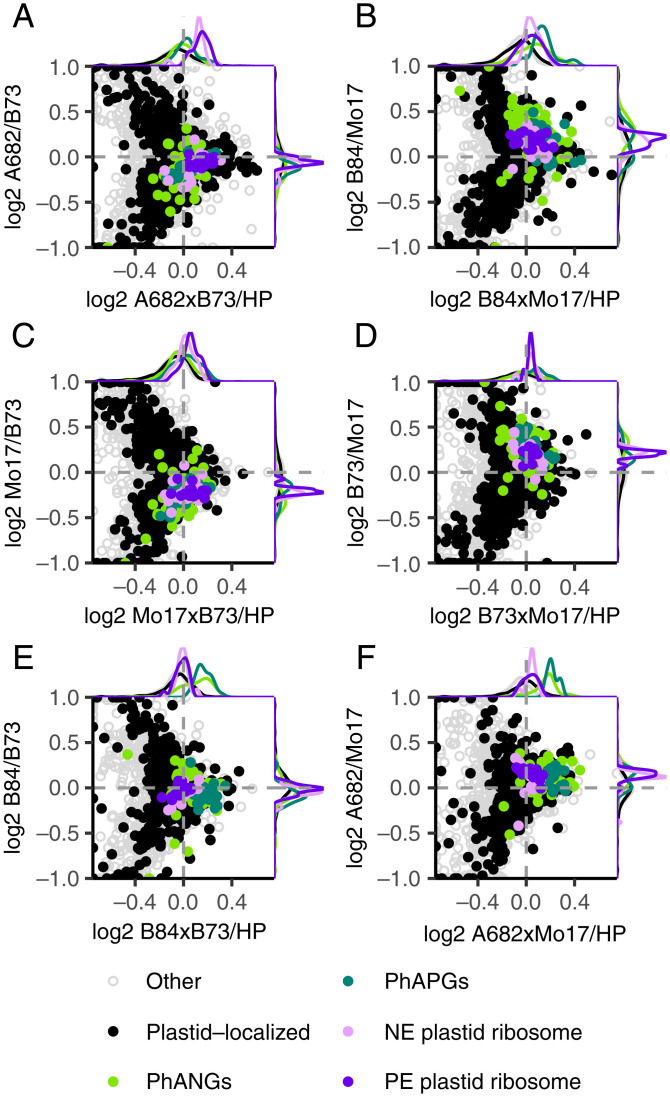
Volcano plots displaying plastid-localized proteins in seedling leaves of six hybrids relative to HP levels. Hybrids represented in *A*–*F* are ordered from greatest plant height heterosis (*A*) to least plant height heterosis (*F*). Points to the left or right of the vertical dotted lines correspond to proteins that are below HP or above HP, respectively. PhANGs, PhAPGs, plastid-encoded (PE) plastid ribosomes, and nuclear-encoded (NE) plastid ribosomes are color-coded.

To evaluate whether the differences in the chloroplast proteome of hybrids are caused by the nuclear or chloroplast genome, we examined the proteome of the reciprocal hybrid, Mo17×B73 (the female parent is listed first). We observed the same differential expression patterns, indicating that the patterns are caused by combining the nuclear genomes, with little or no effect due to maternal inheritance of the plastid genome (Fig. 2 *C* and *D*).

To determine whether the observations were specific for hybrids made between B73 and Mo17, we examined additional hybrids made using the inbreds B84 and A682. Inbreds B73 and B84 are related members of the Stiff Stalk pool of germplasm. Inbreds Mo17 and A682 are part of the Non-Stiff Stalk pool. Hybrids made from crosses between these pools have strong heterosis, in contrast to hybrids made from crosses within each pool (16). The patterns seen with B73 and Mo17 were repeated in the other hybrids, with interesting quantitative variations. The highest overexpression of the PhANG/PhAPG proteins was observed in hybrids with the least heterosis (Fig. 2 *E* and *F*). In contrast, the plastid ribosomal proteins were expressed at substantially above HP levels in the hybrid with the greatest heterosis and at or below HP levels in the hybrids with low levels of heterosis (Fig. 2).

We additionally analyzed nine hybrids made by back-crossing recombinant inbred lines (RILs) to both of the RIL parents, B73 and Mo17. The RIL hybrids were ∼50% as heterozygous as the parent hybrid and were selected based on their varied levels of plant height heterosis (Datasets S3 and S4). We examined collectively these 15 hybrids and their parents, including the RIL hybrids and the 6 hybrids described above. As a measure of trait heterosis, we utilized plant height, which is correlated with grain yield (17). We calculated the Pearson’s correlations between expression heterosis (hybrid expression level/MP expression level) and plant height heterosis (hybrid height/MP height). It is important to note that there was no correlation between expression levels and plant height per se; only the hybrid/MP values for expression and height were correlated. This underscores the specificity of our biomarkers for the trait of heterosis.

**Fig. 3.**
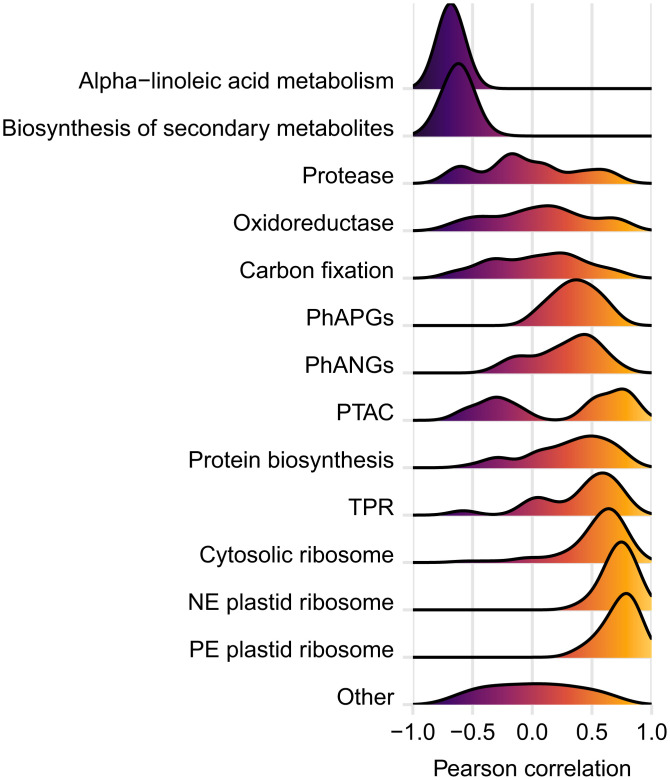
Density curves of Pearson correlations between protein expression heterosis and plant height heterosis in the combined RIL and six hybrids datasets.

The individual Pearson correlations between expression heterosis levels of 581 proteins and plant height heterosis was greater than 0.5 (Dataset S5). This set of proteins was enriched for the terms ribosome (93 proteins), chloroplast (48 proteins), protein biosynthesis (20 proteins), photosynthesis (11 proteins), plastid chromosome (5 proteins), and tetratricopeptide repeat (TPR; 12 proteins) (Fig. 3). Of the top 1.5% of proteins whose expression heterosis was most correlated with plant height heterosis, 18 out of 54 were from the plastid ribosome. Pearson’s correlations of 0.79 to 0.91 were observed for these proteins, half of which were nuclear-encoded, and half were plastid-encoded. Correlations were most positive for the plastid ribosomal proteins, slightly less positive for the cytosolic ribosomal proteins, and moderately positive for the PhANG/PhAPG proteins (Fig. 3). There were 413 proteins with a Pearson’s correlation less than −0.5; these were most strongly enriched for oxidoreductase (39 proteins), biosynthesis of secondary metabolites (63 proteins), protease (18 proteins), biosynthesis of antibiotics (32 proteins), and alpha-linoleic acid metabolism (7 proteins) terms (Fig. 3). Statistical analyses for the correlations of protein groups shown in Fig. 1 are provided in Dataset S2.

### Discordance between Transcript and Protein Expression Levels.

The proteome differences between chloroplasts of the hybrid and its inbred parents were not mirrored in their nuclear transcriptomes (*SI Appendix*, Fig. S4) (18, 19). For example, most nuclear-encoded, plastid ribosomal subunit transcripts were higher in Mo17 than in B73 (*SI Appendix*, Fig. S4*C*), in contrast to their proteins, which were higher in B73 (Fig. 1*C*). The elevated levels of these proteins in B73 appear to be dominant traits because these proteins were expressed above MP levels in the hybrid (Fig. 1*B*). Expression at low levels for these transcripts in B73 also appears to be dominant because they were expressed below MP levels in the hybrid. Expression levels of the PhANG proteins were above HP, with B73 as the HP. Their transcripts showed the same patterns, unlike the plastid ribosomal subunit transcripts. Transcript-expression heterosis patterns in the seedling leaf were not reflected in the mature leaf blade tissues (*SI Appendix*, Fig. S5*A*). However, relative expression levels between B73 and Mo17 were similar between the two developmental stages (*SI Appendix*, Fig. S5*B*).

Correlations between plant height heterosis and plastid ribosomal proteins were high. However, correlations for their transcripts were lower (*SI Appendix*, Fig. S6). Conversely, correlations for the PhANG transcripts were stronger than those of their proteins. Transcripts for the cytosolic ribosome had no correlation with plant height heterosis, unlike their proteins.

Overall, 640 transcripts were expressed above HP, with functional enrichment for circadian rhythm accounting for 7 transcripts; the remaining transcripts had no functional enrichment. With a few exceptions, most circadian-related transcripts were expressed above MP levels (*SI Appendix*, Fig. S7*A*), while expression heterosis of these transcripts varied in their correlations with plant height heterosis (*SI Appendix*, Fig. S7*B*). For example, phytochrome B transcripts were positively correlated, while PHYTOCHROME-INTERACTING FACTOR 3 transcripts were negatively correlated. The transcripts for most sigma factors were expressed above MP levels and were positively correlated with plant height heterosis (*SI Appendix*, Table S1). There were 40 transcription factors expressed above HP, including 5 ET-responsive factors, 4 HOX-domain proteins, 1 auxin-responsive factor, and the circadian regulator, LHY. Conversely, 517 transcripts were expressed below LP, with functional enrichment for metabolic pathways, especially biosynthesis of amino acids. Only one transcription factor, PHD16, was expressed below LP.

### Expression Heterosis Is Phenocopied by an *acs* Mutant.

Mutations in two of the maize genes encoding 1-aminocyclopropane-1-carboxylate (ACC) synthase have been functionally characterized (*Zmacs2* and *Zmacs6*) and were shown to have decreased endogenous ET levels (20–23). Transcripts for ZmACS2, the ACO2 homolog, and the SAM1/2 homolog were found to be expressed substantially below MP levels (Dataset S6), whereas the ZmACS6 transcript was not detected. Several isoforms of enzymes predicted to be involved in ET biosynthesis were also expressed significantly below MP levels, although it is unclear if reduced expression of these enzymes would cause a reduction in ET levels.

**Fig. 4.**
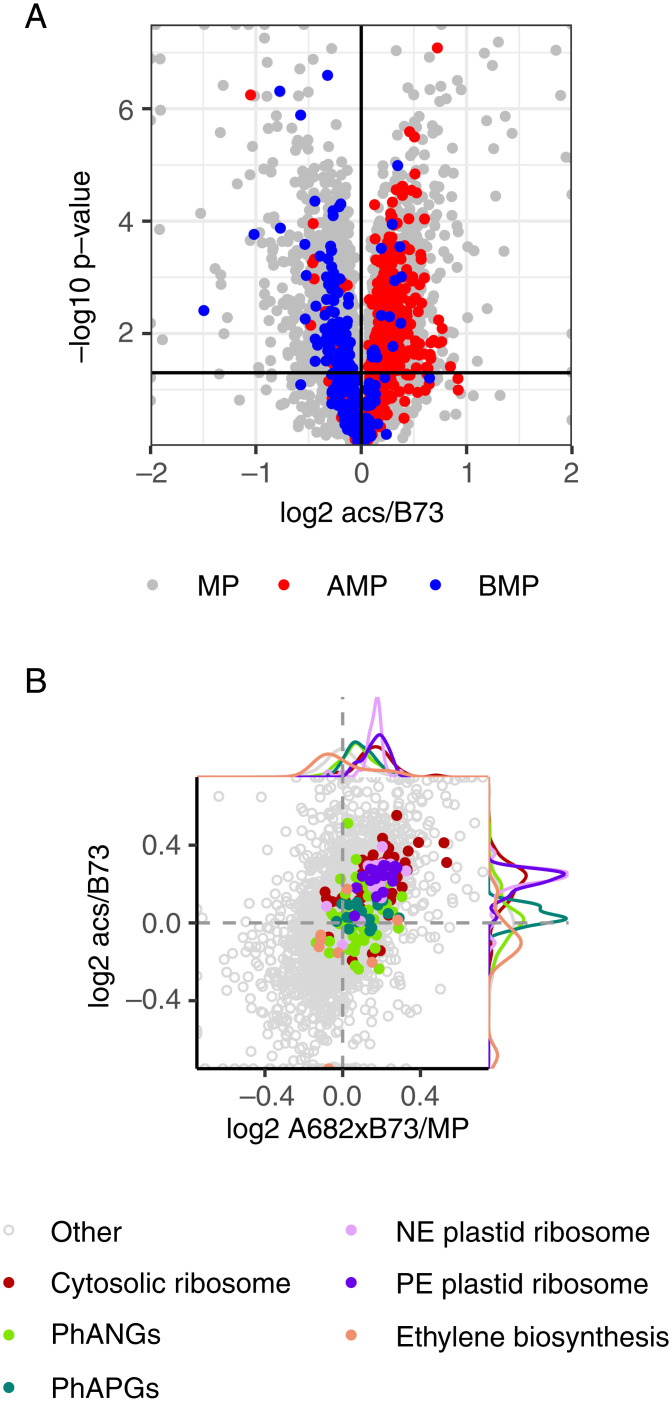
(*A*) Volcano plot of protein expression in the *acs2-6* double mutant in B73 background relative to B73. Each data point represents the mean of five biological replicates. Colors correspond to their expression in the hybrid with greatest plant height heterosis, A682×B73, designated as MP, above MP (AMP), or below MP (BMP). (*B*) Plastid-localized proteins in seedling leaves of the *acs2-6* double mutant in B73 background relative to B73, compared to expression in A682x×B73 relative to MP. Points to the left or right of the vertical dotted line correspond to proteins that are below MP or above MP, respectively. Proteins above or below the horizontal dotted line correspond to proteins that are overexpressed or underexpressed in the mutant, respectively. PhANGs, PhAPGs, plastid-encoded (PE) plastid ribosomes, nuclear-encoded (NE) plastid ribosomes, and ET biosynthesis proteins are color-coded.

Proteomic analysis of the ET biosynthesis double mutant *Zmacs2/6* in the B73 genetic background (20) revealed that the mutant phenocopied the hybrid molecular phenotype. Proteins expressed above or below MP in A682×B73 (the hybrid with the greatest heterosis) were expressed similarly in the mutant relative to the inbred, B73 (Fig. 4*A*). Protein levels of most PhANGs/PhAPGs and plastid ribosome subunits were elevated in the mutant at equal proportions, as in A682×B73 (Fig. 4*B*). The exceptions were two ATP synthase subunits and two plastid ribosomal proteins, which were significantly above MP in the hybrid, but unchanged in the mutant. Statistical analyses for the differential expression of protein groups shown in Fig. 4*B* are provided in Dataset S2. Expression levels of jasmonic acid (JA) biosynthesis enzymes were reduced in the *Zmacs2/6* mutant relative to B73 and in hybrids relative to MP (*SI Appendix*, Fig. S8). Of the 538 proteins expressed above MP in A682×B73, only 18 were repressed in *Zmacs2/6*; these were enriched for biosynthesis of secondary metabolites and also included three PLASTID TRANSCRIPTIONALLY ACTIVE CHROMSOME (PTAC) proteins. Of the 285 proteins expressed below MP in A682×B73, only 15 were elevated in *Zmacs2/6*; no functional enrichment was found for these.

## Discussion

Most plastid-localized proteins were expressed at MP levels in the hybrid B73×Mo17. Nonadditive expression patterns were observed for a minority of proteins and RNAs. Photosynthesis-related proteins and plastid ribosomal proteins represented the major groups expressed above HP and MP levels, respectively. For both groups, the nuclear-encoded and plastid-encoded subunits were expressed similarly. Elevated expression of photosynthetic proteins in hybrids is diurnally regulated, with highest levels in the morning (13), which corresponds to the sampling time in our experiments. Many transcription factors were also expressed above HP. Proteins and transcripts expressed below LP were enriched most strongly for amino acid biosynthesis, consistent with previous measurements of hybrid metabolite levels (13). We observed that circadian-related genes were enriched in the transcripts expressed above HP, consistent with previous reports of an altered circadian rhythm in hybrids (9, 10, 24, 25). Temporal epigenetic activation and repression of the circadian clock regulator, CCA1, eliminate the immunity-growth tradeoff in hybrids (25). Consistent with this finding, we observe above MP *CCA1* expression in the B×M hybrid (*SI Appendix*, Fig. S7). Translation can be influenced by circadian rhythms (26), which may account for the altered translation patterns we observed in hybrids.

While developmental differences between hybrids and inbreds could result in differences in expression levels, we controlled for such differences as much as possible by staggering planting dates to ensure that all plants were at the same developmental stage at the time of sampling, as well as by harvesting all tissues within 15 min for each experiment. The proteomes of inbreds and hybrids within an experiment were nearly as similar to each other as to their biological replicates (*SI Appendix*, Fig. S3), indicating that the plants were sampled at the same developmental stage. To further ensure that the observed differences between inbreds and hybrids did not arise from a sampling mismatch between plants growing at different rates, we sampled from both juvenile and adult leaves. As expected, the different developmental stages of these tissues caused large differences between their proteomes (*SI Appendix*, Fig. S3). Nevertheless, they displayed the same differences between inbreds and hybrids (*SI Appendix*, Fig. S2*A*), indicating that the differences are not stage-specific or caused by sampling issues.

We hypothesize that plastid ribosome levels were positively correlated with plant height heterosis because ribosomes produce the abundant proteins required for photosynthesis and for enabling proteostasis of plastid-localized, metabolic enzymes (27). It was surprising that the PhAPG and PhANG proteins had a lower correlation to trait heterosis. We hypothesize that damage to photosynthetic proteins in the light causes the accumulation of a substantial pool of inactive proteins, and our methods do not distinguish active from inactive forms. This could contribute to the unusually high levels of PhAPG and PhANG proteins in hybrids, and it could reduce the correlations between their protein and mRNA levels. The cytoplasmic ribosome was nearly as well correlated as the plastid ribosome and is also likely to contribute to heterosis traits. Subunits of the cytoplasmic ribosome are expressed above MP in the maize seminal root (28). In yeast, proteome reallocation to ribosomes enables faster growth in rich medium (29). Elevated photosynthetic capacity may be important for heterosis, even though it does not appear to be quantitatively coupled to the levels of heterosis. Hybrids made from inbreds of the same heterotic pool expressed the PhANG and PhAPG proteins above HP levels, whereas PhANG transcripts were higher in hybrids made from crosses between heterotic pools. Consequently, PhANG transcripts were quantitatively associated with plant height heterosis, while their protein levels were not. Nevertheless, hybrids have greater photosynthetic capacity than their parents, and this may be necessary to produce the greater biomass and yield of hybrids (10).

Other groups of proteins with positive correlations to heterosis included PTAC proteins, which are essential for accumulation of the plastid‐encoded polymerase complex (30), and protein biosynthesis-related proteins, consisting of elongation and initiation factors. Elevated expression of both of these groups of proteins in the hybrid may contribute to the hybrid-specific expression patterns of the chloroplast- and nuclear-encoded proteins. TPR proteins were also enriched in the positively correlated group, and some of these are known to mediate protein–protein interactions and play roles in stress and hormone signaling (31). Additionally, transcripts for most sigma factors were overexpressed in the hybrid and were positively correlated with plant height heterosis. We hypothesize that nuclear-encoded, plastid sigma factors are causing plastid gene transcript levels to be greater in the hybrids. The consequently higher levels of plastid-encoded proteins may enable nuclear-encoded subunits of the same complexes to be stabilized at higher levels, accounting for the observed differences between inbreds and hybrids.

Groups of proteins enriched among those with negative correlations included oxidoreductase, consisting of several dehydrogenases, lipoxygenases, and one of the ET biosynthetic enzymes, ACC oxidase. Proteases were negatively correlated. The two most negatively correlated groups were biosynthesis of secondary metabolites and alpha-linoleic acid metabolism, consistent with the proposed tradeoff between stress responses and plant growth (32). Carbon-fixation-related proteins had a mixed pattern of expression differences in the hybrid and overall were neither positively nor negatively correlated with plant height heterosis. Others report that hybrids are associated with either elevated levels of photosynthetic proteins (hybrid ZD909) or stress proteins (hybrid ZD808), but not both (33).

Our results indicate that the physiology of heterosis may be conserved between monocots and dicots. ET biosynthetic enzyme gene expression was repressed in maize hybrids, as was reported for *Arabidopsis* hybrids (9). ACS expression is down-regulated by CCA1 and PIF5 in *Arabidopsis* hybrids (9). Transcripts for both CCA1 and PIF5 were expressed above MP in the B×M hybrid, which could account for reduced expression of ET biosynthesis enzymes if their regulation is conserved. Additionally, the ACO2 homolog is polymorphic between B73 and Mo17, which may account for its altered expression in the hybrid. Reduced expression of ET biosynthetic enzymes caused by the *Zmacs2/6* mutation in B73 phenocopied the heterosis expression levels of chloroplast proteins. Traits of the maize *acs2* and *acs6* mutants are known to resemble those of hybrids; these include transpiration, stomatal closure, carbon dioxide assimilation, and elevated levels of foliar chlorophyll, Rubisco, and soluble protein (20). JA biosynthetic enzyme levels were repressed in both the hybrid and in the *Zmacs2-6* mutant, indicating that JA biosynthesis may be regulated by ET. Interestingly, the JA biosynthetic pathway was enriched in the proteins that were negatively correlated with plant height heterosis, suggesting a potential role for reduced JA biosynthesis. The most notable differences between A682×B73 (the hybrid with the greatest heterosis) and the *Zmacs2-6* mutant were expression levels of three PTAC proteins, which were elevated in the hybrid and repressed in the mutant. Levels of these plastid RNA polymerase protein subunits were positively correlated with plant height heterosis.

The discrepancy between accumulation patterns of transcripts and their proteins argues that posttranscriptional mechanisms are selectively regulating protein levels in the inbreds and their hybrids. Expression of plastid-encoded proteins can be under translational regulation. For example, the PSII reaction center protein, D1, participates in an autoregulatory mechanism, in which it represses its own translation unless it becomes incorporated into the PSII complex (34). Such mechanisms ensure stoichiometry between nuclear- and plastid-encoded subunits. Expression of nuclear genes encoding ribosomal proteins also is under translational regulation (35). Expression heterosis of these proteins was highly correlated with plant height heterosis.

Relative expression between B73 and Mo17 of PhANG transcripts and proteins is similar between the seedling leaf and mature leaf blade, with B73 as the HP. In contrast, B73 is the HP for plastid ribosomal proteins in the seedling leaf, but not the leaf blade, while Mo17 is the HP for their transcripts in both tissues. It is unclear how the seedling leaf of B73 accumulates more of each ribosomal protein from substantially lower transcript levels than in Mo17. Developmental and circadian patterns of expression can uncouple transcripts from their proteins (36), and differential translation of mRNAs can occur throughout the day (26). Mechanisms such as these may play a role in the differential protein expression of inbreds and their hybrids. While expression heterosis patterns were similar between seedling leaf and mature leaf blade tissues for proteins, RNA expression heterosis patterns differed between the two tissues. Consistent with this observation, previous studies report minimal evidence for nonadditive transcript-expression patterns maintained across multiple tissues in the hybrid (37). Our results show that proteome and transcriptome expression patterns for sets of genes are consistent for the genes within a protein complex and can differ between complexes and between developmental stages. If there are regulators that coordinate these above-MP expression patterns, they are specific for either the plastid ribosome or for the PhANGs. Such regulators may play differential roles in the especially strong expression of PhANGs/PhAPGs in hybrids from inbreds of the same heterotic group (Fig. 2 *E* and *F*) and in the especially strong expression of the plastid ribosomal proteins in the ET biosynthesis mutant (Fig. 4*B*). Proteome dominance has been observed in the nonphotosynthetic immature ear of the maize hybrid, ZD909 (7). The authors found that many enzymes involved in carbon and nitrogen assimilation were expressed at HP levels. In *Arabidopsis*, transcripts for cell division are more highly expressed in one parent, and in the other parent, transcripts for photosynthesis are higher. The hybrid expressed both sets above MP levels, and the combination of the two pathways may be important for heterosis (38). Further work is needed to understand the mechanisms of expression dominance and the roles it may play in heterosis.

## Materials and Methods

### Plant Growth and Sampling.

Plant materials were all grown in growth chambers for collection of seedling leaf tissues and grown in the field for collection of the mature leaf blade tissues. At least three biological replicates were used for each genotype. All tissues were harvested between 9 and 10 AM. Within an experiment, all sampling was performed within a 15-min period to minimize circadian differences among samples. We utilized staggered planting dates to ensure that we had plants all at the same developmental stage on the same date for sampling. Tissue samples were ground in liquid nitrogen, and then portions of the ground tissue were split into the transcriptome or proteome analysis pipeline. Details on the plant materials and sampling and phenotyping are described in *SI Appendix*, *Materials and Methods*.

### RNA-Sequencing Analysis.

Sequence libraries were prepared by using the standard TruSeq Stranded mRNA library protocol and sequenced on NovaSeq 150-bp paired-end S4 flow cell to produce at least 20 million reads for each sample. All transcriptome data were submitted to the National Center for Biotechnology Information (NCBI) Sequence Read Archive (SRA) (accession no. PRJNA747924). Counts per million values were calculated (Datasets S7 and S8) and used to calculate averages and ratios. Details and procedures for RNA-sequencing and data analysis are described in *SI Appendix*, *Materials and Methods*.

### Proteomics Data Acquisition and Analysis.

Spectra were acquired on a Q-exactive-HF mass spectrometer (Thermo Electron Corporation). TMT abundances were normalized to the arithmetic mean of the B73 replicates in each run (Dataset S9) and used to calculate averages and ratios. Protein group assignment was based off of the Kyoto Encyclopedia of Genes and Genomes (39), CornCyc (40), and the NCBI (41) (Dataset S10). Details and procedures for proteomics methods and data analysis are described in *SI Appendix*, *Materials and Methods*.

## Supplementary Material

Supplementary File

Supplementary File

Supplementary File

Supplementary File

Supplementary File

Supplementary File

Supplementary File

Supplementary File

Supplementary File

Supplementary File

Supplementary File

## Data Availability

Proteomics data have been deposited in the Mass Spectrometry Interactive Virtual Environment (accession no. MSV000085916), and anonymized transcriptomics data have been deposited in the NCBI SRA (accession no. PRJNA747924). All study data are included in the article and/or supporting information. Previously published data were used for this work (37).

## Change History

December 14, 2021: The footnotes have been updated.
