# Supplementary material for: Plant height heterosis is quantitatively associated with expression levels of plastid ribosomal proteins
Source: Proc Natl Acad Sci U S A. 2021 Nov 15;118(47):e2109332118. doi: 10.1073/pnas.2109332118 (PMC8617506; doi:10.1073/pnas.2109332118)
Supplement: Supplementary File [file pnas.2109332118.sapp.pdf]

PNAS
www.pnas.org

Supplementary Information for
Plant height heterosis is quantitatively associated with expression levels of plastid ribosomal proteins

Devon Birdseye[1], Laura A. de Boer[1], Hua Bai[1], Peng Zhou[2], Zhouxin Shen[1], Eric A. Schmelz[1], Nathan M. Springer[2], Steven P. Briggs[1]

[1] Division of Biological Sciences, University of California, San Diego, La Jolla, CA 92093, USA
[2] Department of Plant and Microbial Biology, University of Minnesota, Saint Paul, MN 55108, USA

*Steven P. Briggs

Email: sbriggs@ucsd.edu

This PDF file includes:

Supplementary Materials and Methods
Figures S1 to S8
Table S1
Legends for Datasets S1 to S10
SI References

Other supplementary materials for this manuscript include the following:

Datasets S1 to S10

Supplementary Materials and Methods

Plant growth and sampling. The seedling leaf and mature leaf blade tissues of B73, Mo17 and B73xMo17 were collected in May of 2016 in St Paul, Minnesota, while tissues from the six hybrids and RIL hybrids experiments were collected from July to November of 2019 in St Paul, Minnesota. Plants were grown in the field for collection of adult tissues. For seedling tissues, seeds were imbibed for 24 hours in distilled water and grown in growth chambers at 25℃ under 12h light-dark cycles to the V3 stage (day 9) for collection of the V2 leaves. Tissue from at least four plants was pooled for each replicate and the three biological replicates were harvested between 9 and 10 AM in the morning. Within an experiment, all sampling was performed within a 15-minute period to minimize circadian differences among samples. We utilized staggered planting dates to ensure the plants were all at the same developmental stage on the same date for sampling. Tissue samples were ground in liquid nitrogen and then portions of the ground tissue were split into the transcriptome or proteome analysis pipeline. For the *acs2/6* mutant and B73 seedling leaf samples, seeds were imbibed for 48 hours and manually examined to select uniformly germinating seedlings based on radical emergence and the lack of mold. They were then grown in growth chambers at 25°C under 12h light-dark cycles to V3 stage (day 10), and the V2 leaf was harvested at 10 AM for 5 biological replicates of each genotype.

RNA-Seq analysis. Sequence libraries were prepared using the standard TruSeq Stranded mRNA library protocol and sequenced on NovaSeq 150bp paired end S4 flow cell to produce at least 20 million reads for each sample. Both library construction and sequencing were done in the University of Minnesota Genomics Center. All transcriptome data is submitted to NCBI SRA (accession PRJNA747924). Sequencing reads were then processed through the nf-core RNA-Seq pipeline (1) for initial QC and raw read counting. In short, reads were trimmed by Trim Galore! (https://github.com/FelixKrueger/TrimGalore) and aligned to the B73 maize reference genome (Zm-B73-REFERENCE-GRAMENE-4.0) (2) using the variant-aware aligner Hisat2 (3) which also takes 90 million common variants to account for mapping bias. Uniquely aligned reads were then counted per feature by featureCounts (4). Raw read counts were then normalized by library size and corrected for library composition bias using the TMM normalization approach (5) to give CPMs (Counts Per Million reads) for each gene in each sample, allowing direct comparison across samples (Supplemental Tables 6 and 7). Hierarchical clustering, principal component analysis and t-SNE clustering were used to explore sample cluster patterns and to remove or substitute bad replicates.

Proteomics Sample Preparation. Ground tissue powders were suspended in extraction buffer (8M Urea/100mM Tris/5mM Tris(2- carboxyethyl)phosphine (TCEP)/phosphatase inhibitors, pH 7). Proteins were precipitated by adding 4 volumes of cold acetone and incubated at 4oC for 2 hours. Samples were centrifuged at 4,000xg, 4oC for 5 minutes. Supernatant was removed and discarded. Proteins were re-suspended in Urea extraction buffer (8M Urea/100mM Tris/5mM TCEP/phosphatase inhibitors, pH 7) and precipitated by cold acetone one more time. Protein pellets were washed by cold methanol to further remove non-protein contaminants. Protein pellets were suspended in extraction buffer (8M Urea/100mM Tris/5mM TCEP/phosphatase inhibitors, pH 7). Proteins were first digested with Lys-C (Wako Chemicals, 125-05061) at 37oC for 15 minutes. Protein solution was diluted 8 times to 1M urea with 100mM Tris and digested with trypsin (Roche, 03 708 969 001) for 4 hours. Digested peptides were purified on a Waters Sep-Pak C18 cartridges, eluted with 60% acetonitrile. TMT-10 labeling was performed in 50% acetonitrile/150mM Tris, pH7. TMT labeling efficiency was checked by LC-MS/MS to be greater than 99%. Labeled peptides from different samples were pooled together for 2D-nanoLC-MS/MS analysis. An Agilent 1100 HPLC system was used to deliver a flow rate of 600 nL min-1 to a custom 3-phase capillary chromatography column through a splitter. Column phases were a 20 cm long reverse phase (RP1, 5 μm Zorbax SB-C18, Agilent), 6 cm long strong cation exchange (SCX, 3 μm PolySulfoethyl, PolyLC), and 20 cm long reverse phase 2 (RP2, 3.5 μm BEH C18, Waters), with the electrospray tip of the fused silica tubing pulled to a sharp tip (inner diameter <1 μm). Peptide mixtures were loaded onto RP1, and the 3 sections were joined and mounted on a

custom electrospray adapter for on-line nested elutions. Peptides were eluted from RP1 section to SCX section using a 0 to 80% acetonitrile gradient for 60 minutes, and then were fractionated by the SCX column section using a series of 20 step salt gradients of ammonium acetate over 20 min, followed by high-resolution reverse phase separation on the RP2 section of the column using an acetonitrile gradient of 0 to 80% for 150 minutes.

Proteomics data acquisition. Spectra are acquired on a Q-exactive-HF mass spectrometer (Thermo Electron Corporation, San Jose, CA) operated in positive ion mode with a source temperature of 275 °C and spray voltage of 3kV. Automated data-dependent acquisition was employed of the top 20 ions with an isolation windows of 1.0 Da and collision energy of 30. The mass resolution is set at 60,000 for MS and 30,000 for MS/MS scans, respectively. Dynamic exclusion is used to improve the duty cycle.

Proteomics data analysis. The raw data was extracted and searched using Spectrum Mill vB.06 (Agilent Technologies). MS/MS spectra with a sequence tag length of 1 or less were considered to be poor spectra and were discarded. The remaining high quality MS/MS spectra were searched against a maize protein database including maize B73 (Zm-B73-REFERENCE-GRAMENE-4.0) and Mo17 (Zm-Mo17-REFERENCE-CAU-1.0) reference genomes (2, 6). A 1:1 concatenated forward-reverse database was constructed to calculate the false discovery rate (FDR). There were 435,212 protein sequences in the final protein database. Search parameters were set to Spectrum Mill's default settings with the enzyme parameter limited to full tryptic peptides with a maximum mis-cleavage of 1. Cutoff scores were dynamically assigned to each dataset to obtain the false discovery rates (FDR) of 0.1% for peptides, and 1% for proteins. Proteins that share common peptides were grouped using principles of parsimony to address protein database redundancy. Total TMT-10 reporter intensities were used for relative protein quantitation. Peptides shared among different protein groups were removed before TMT quantitation. Isotope impurities of TMT-10 reagents were corrected using correction factors provided by the manufacturer (Thermo). Median normalization was performed to normalize the protein TMT-10 reporter intensities in which the log ratios between different TMT-10 tags were adjusted globally such that the median log ratio was zero.

Proteomics data deposition. The raw spectra for the proteome data have been deposited in the Mass Spectrometry Interactive Virtual Environment (MassIVE) repository (massive.ucsd.edu/ProteoSAFe/static/massive.jsp) (accession ID MSV000085916). FTP download link before publication: ftp://MSV000085916@massive.ucsd.edu; FTP download link after publication: ftp://massive.ucsd.edu/MSV000085916/.

Expression analysis. For protein analyses, tandem-mass tag (TMT) abundances were normalized to the arithmetic mean of the B73 replicates in each run (Supplemental Dataset 9). For RNA analysis of the original seedling leaf and leaf blade, previously published counts per million (CPM) values were used (7) For six hybrid and RIL hybrid RNA analyses, CPM values were used (Supplemental Datasets 7 and 8 for six hybrids and RIL hybrids, respectively). For each experiment, genes or proteins were removed if they were not detected in all biological replicates. The arithmetic mean of the biological replicates was then calculated and used to calculate hybrid/mid-parent and parent/parent ratios. The log base 2 was then calculated for each ratio. A T-test was used to calculate p-values. Data was manipulated using the R packages dplyr (8), plyr (9), tidyr (10), reshape2 (11), stringr (12), and data.table (13). Functional enrichment performed using The Database for Annotation, Visualization and Integrated Discovery (DAVID) (14, 15). Scatterplots and volcano plots were generated using ggplot2 (16), density plots were generated using cowplot (17), and plots were assembled into paneled figures using patchwork (18). KEGG maps were generated using pathview (19), with the default settings in which the sum is calculated for each enzyme for expression maps and the absolute maximum calculated for the Pearson correlation map. Colors for CCA1 on the circadian rhythm maps were added manually as they were not mapped by KEGG. For correlation analyses, the Pearson correlations (Supplemental Dataset 5) were calculated from the hybrid/mid-parent expression ratios (Supplemental Dataset 1) to the hybrid/mid-parent plant height ratios (Supplemental Dataset 4)

and plotted using ggridges (20). Z-tests were performed using BSDA (21). All source codes used for expression analyses are available on Github: https://github.com/devonbirdseye/HeterosisManuscript

Protein group assignment. The plastid proteome was defined according to a previous report (22). PhANGs were defined as the proteins in known chloroplast complexes using KEGG maps zma00195, excluding the electron transport proteins, zma00196, and CornCyc reactions 1.97.1.12 and 1.10.3.9 (23, 24), all of which were downloaded on February 26, 2020. The PhAPGs were defined as the plastid-encoded proteins with NCBI annotations (25) as PSI, PSII, cytochrome b6f, and ATP synthase. Nuclear-encoded plastid ribosomal proteins were defined as those containing “30S” or “50S” in their NCBI annotation (25) as well as “plastid” or “chloroplast” in their maize-GAMER GO annotations (26), with term descriptions retrieved using the R package AnnotationDbi (27). Cytosolic ribosomal proteins were defined as those containing “40S” or “60S” in their NCBI annotation, except for those containing “mitochondrial” or “biogenesis”. Ethylene biosynthetic proteins were defined using CornCyc reactions 2.5.1.6, 4.4.1.14 and 1.14.17.4 (24), downloaded on May 5, 2020. All accession conversions to v4 were made using the MaizeGDB accession conversion table, downloaded on May 18, 2020 (24). Proteins involved in carbon fixation, biosynthesis of secondary metabolites, and alpha-linoleic acid metabolism were defined using KEGG maps zma00710, zma01110, and zma00592, respectively. TPR and PTAC proteins were defined as those containing “TPR” and “plastid transcriptionally active” in their NCBI annotation (25), respectively. Oxidoreductase and protease proteins were defined as those with the term GO:0016491 and GO:0008233 in their maize-GAMER GO annotations (26), respectively. Protein biosynthesis proteins were defined as those containing “elongation factor” or “initiation factor” in their NCBI annotation (25). A complete list of gene group assignments and their sources is provided in Supplemental Dataset 10.

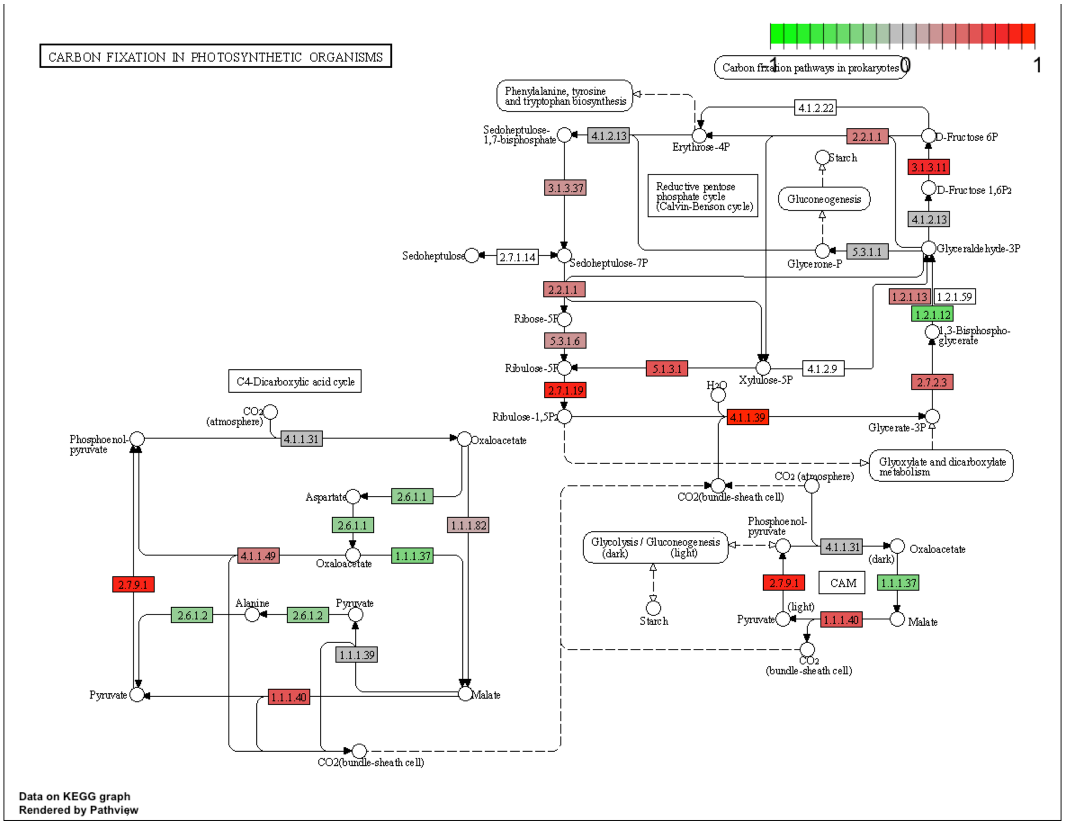

Fig. S1. Expression of carbon fixation enzymes in the hybrid B73xMo17 relative to its mid-parent level.

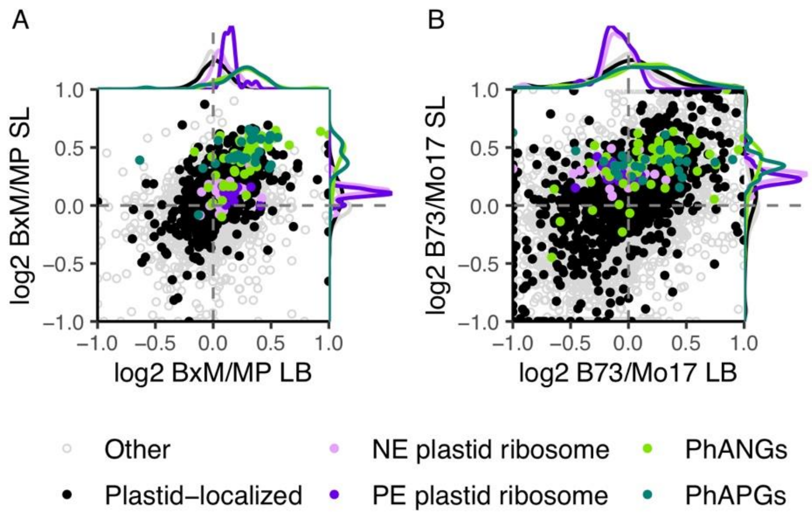

Fig. S2. Comparison of expression of the significantly non-additive proteins in seedling leaf (SL) versus leaf blade (LB) tissue. A compares hybrid/mid-parent values; B compares parent/parent values. Points to the left of the vertical dotted line correspond to proteins that are below mid-parent (A) or Mo17 high-parent (B) in the leaf blade; points to the right of the vertical dotted line correspond to proteins that are above mid-parent (A) or B73 high-parent (B) in the leaf blade. Points below the horizontal dotted line correspond to proteins that are below mid-parent (A) or Mo17 high-parent (B) in the seedling leaf; points above the horizontal dotted line correspond to proteins that are above mid-parent (A) or B73 high-parent (B) in the seedling leaf. Photosynthesis-associated Nuclear Genes (PhANGs), Photosynthesis-associated Plastid Genes (PhAPGs), Plastid-Encoded (PE) plastid ribosomes, and Nuclear-Encoded (NE) plastid ribosomes are color-coded.

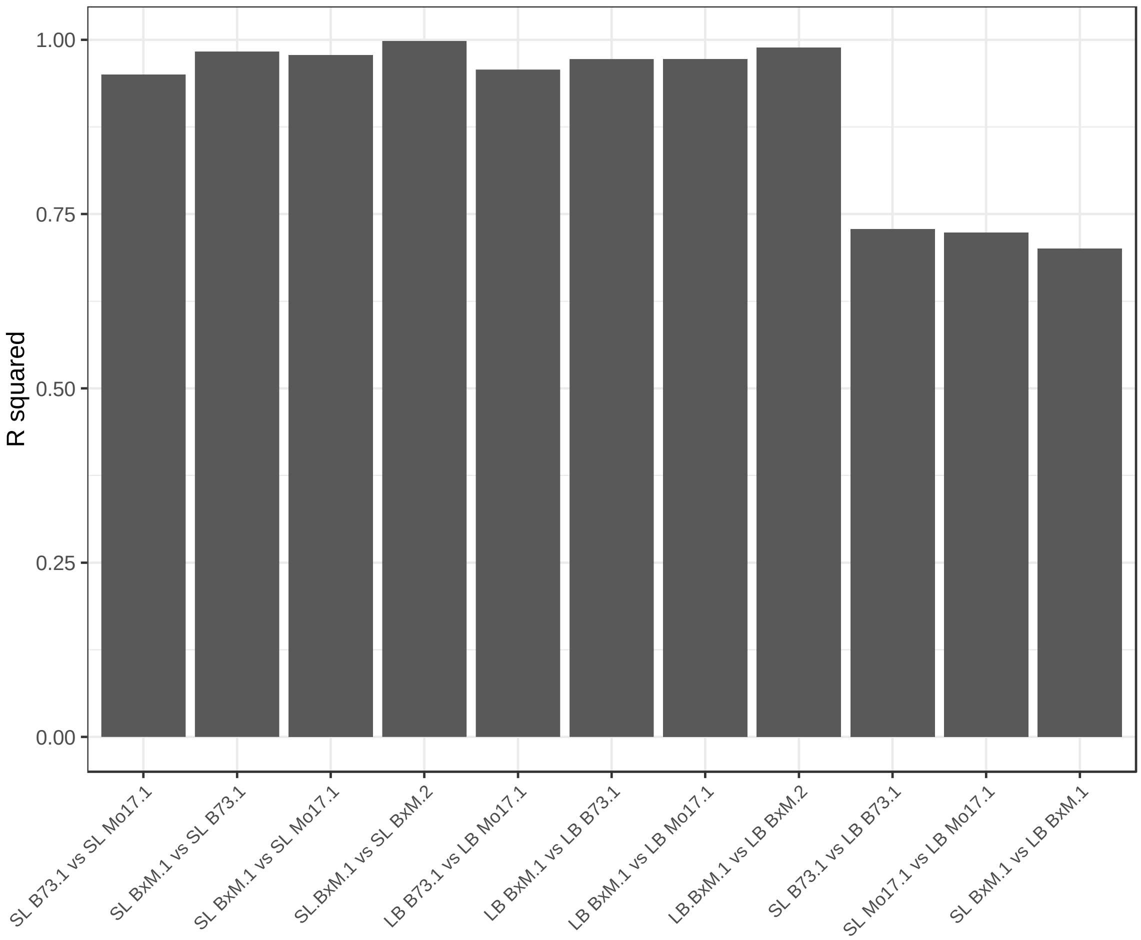

Fig. S3. R-squared values from the comparison of biological replicates of the seedling leaf (SL) and mature leaf blade (LB) tissues.

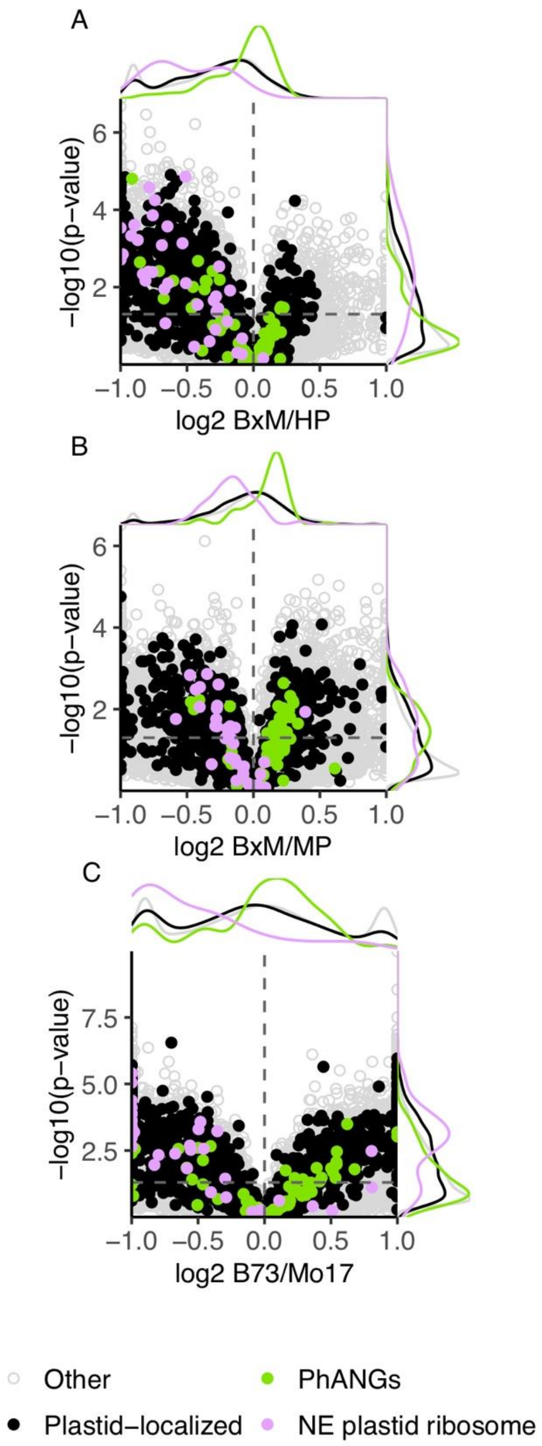

Fig. S4. Volcano plots displaying RNA expression patterns of the most significantly non-additive proteins, representing B73xMo17/high-parent (A), B73xMo17/mid-parent (B), and B73/Mo17 (C). Points to the left of the vertical dotted line correspond to below high-parent (A), below mid-parent (B), or Mo17 high-parent (C) transcripts; points to the right of the vertical dotted line correspond to above high-parent (A), above mid-parent (B), or B73 high-parent (C) transcripts. Photosynthesis-

associated Nuclear Genes (PhANGs), Nuclear-Encoded (NE) plastid ribosome, and are color-coded.

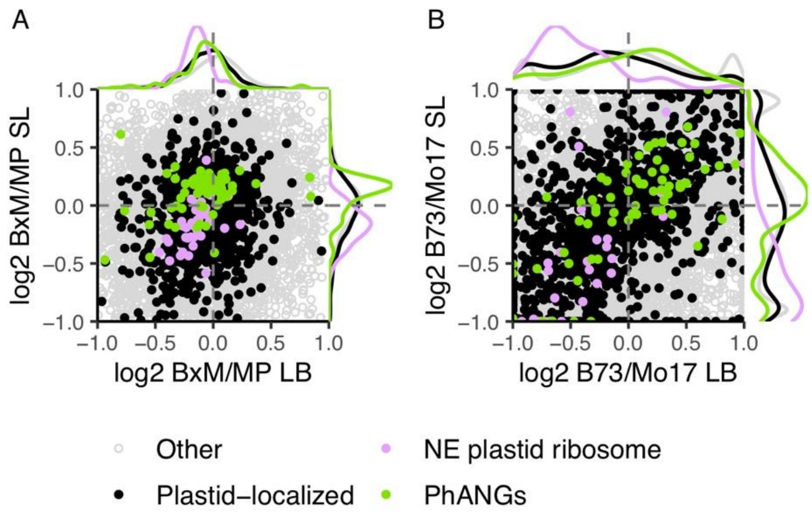

Fig. S5. Comparison of expression of transcripts for the significantly non-additive proteins in seedling leaf (SL) versus leaf blade (LB) tissue. A compares hybrid/mid-parent values; B compares parent/parent values. Points to the left of the vertical dotted line correspond to transcripts that are below mid-parent (A) or Mo17 high-parent (B) in the leaf blade; points to the right of the vertical dotted line correspond to transcripts that are above mid-parent (A) or B73 high-parent (B) in the leaf blade. Points below the horizontal dotted line correspond to transcripts that are below mid-parent (A) or Mo17 high-parent (B) in the seedling leaf; points above the horizontal dotted line correspond to transcripts that are above mid-parent (A) or B73 high-parent (B) in the seedling leaf. Photosynthesis-associated Nuclear Genes (PhANGs) and Nuclear-Encoded (NE) plastid ribosomes are color-coded.

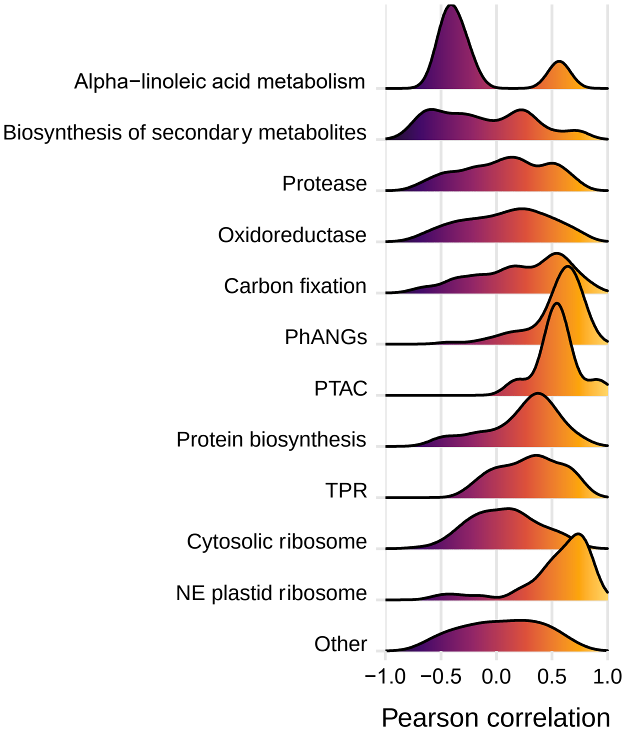

Fig. S6. Density curves of Pearson correlations between RNA expression heterosis and plant height heterosis in the combined RIL and six hybrids datasets.

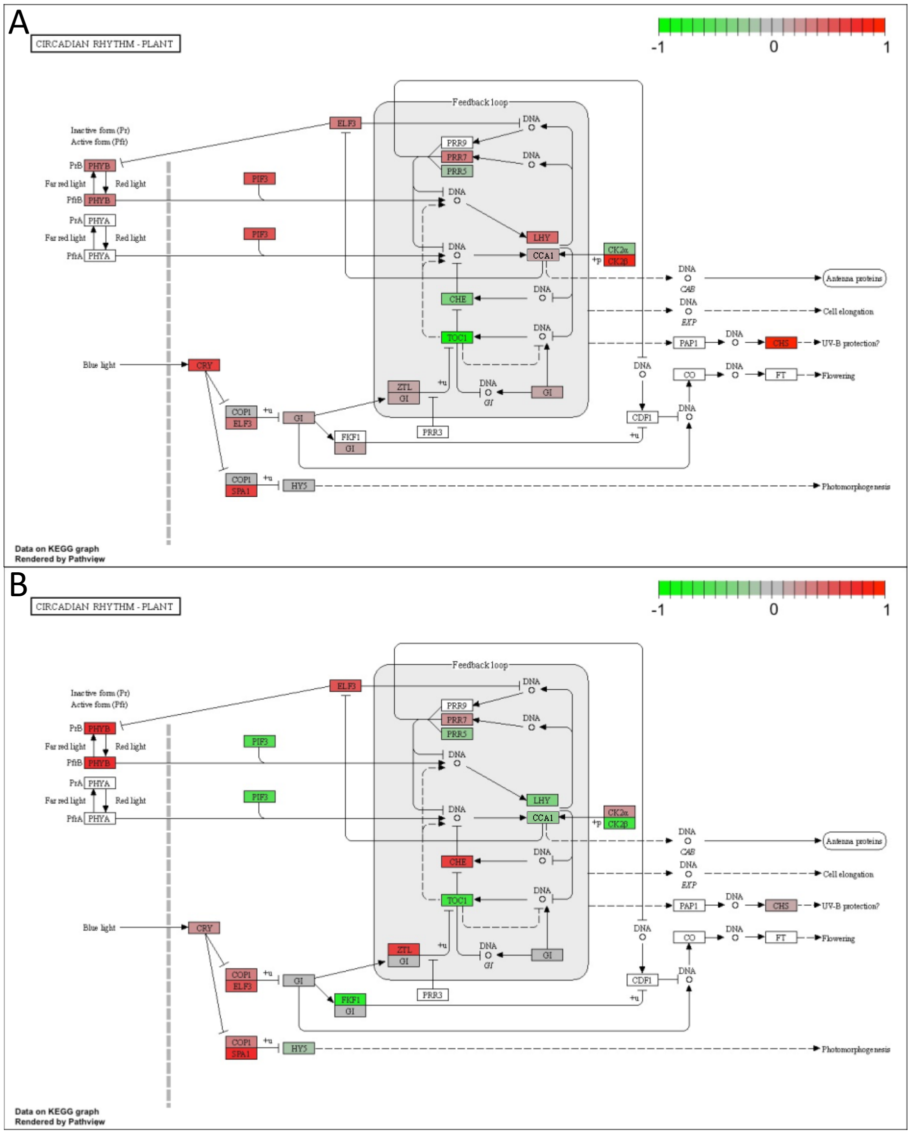

Fig. S7. (A) Expression of circadian rhythm-related transcripts in the hybrid, B73xMo17, relative to its mid-parent level. (B) Pearson correlation of circadian-related transcripts between expression heterosis and plant height heterosis.

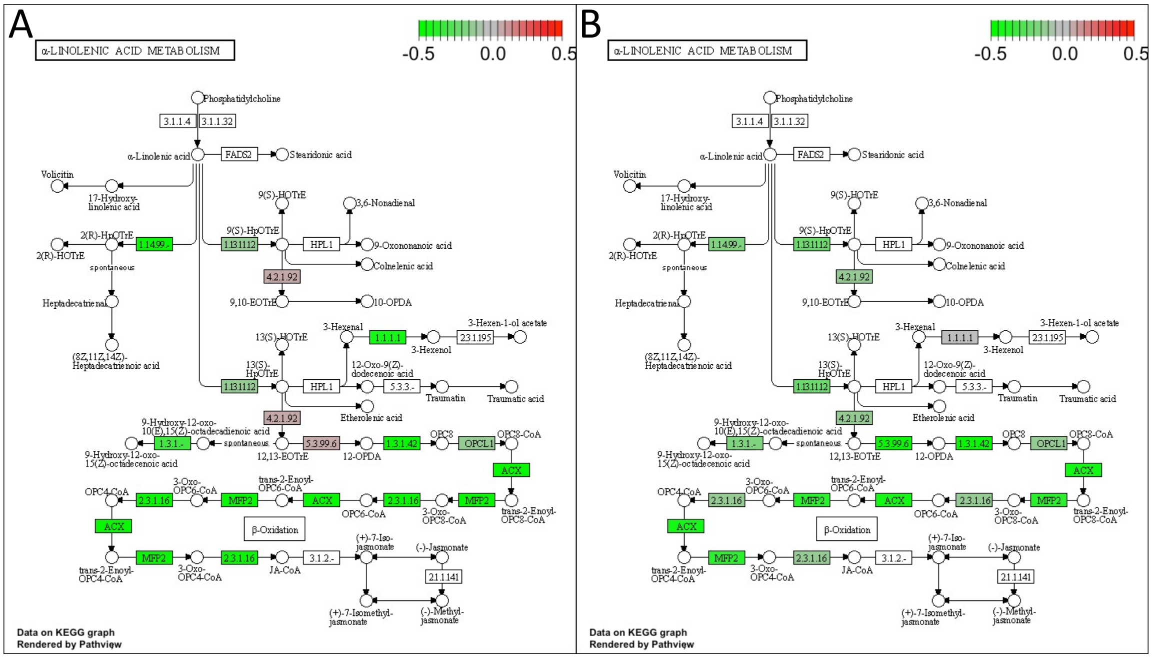

Fig. S8. Expression of JA biosynthesis enzymes in the (A) hybrid B73xMo17 relative to its mid-parent level; and (B) in the ET biosynthesis mutant (acs2-6) compared to its inbred B73 background.

			Protein			RNA		
Gene	Protein	Category	BxM/MP	p-value	cor	BxM/MP	p-value	cor
Zm00001d002490	Plastid-specific 30S ribosomal protein 2	NE plastid ribosome	1.269	0.004	0.41	1.058	0.199	0.80
Zm00001d006196	30S ribosomal protein S31	NE plastid ribosome	0.990	0.559	NA	1.311	0.012	-0.35
Zm00001d008228	50S ribosomal protein 5	NE plastid ribosome	1.142	0.036	NA	0.901	0.069	0.22
Zm00001d008360	50S ribosomal protein L3-2	NE plastid ribosome	0.999	0.966	NA	1.011	0.937	-0.14
Zm00001d010803	50S ribosomal protein L20	NE plastid ribosome	0.943	0.023	NA	0.915	0.565	0.20
Zm00001d011993	50S ribosomal protein L27	NE plastid ribosome	1.155	0.140	0.91	0.916	0.213	0.64
Zm00001d012353	30S ribosomal protein S17	NE plastid ribosome	1.104	0.029	0.86	0.718	0.001	0.81
Zm00001d012998	50S ribosomal protein L17	NE plastid ribosome	1.106	0.061	0.75	0.756	0.003	0.69
Zm00001d014153	50S ribosomal protein L28	NE plastid ribosome	0.977	0.760	NA	NA	NA	NA
Zm00001d014488	50S ribosomal protein L24	NE plastid ribosome	1.072	0.007	0.53	0.785	0.001	0.61
Zm00001d015204	50S ribosomal protein L3-1	NE plastid ribosome	1.111	0.012	0.81	0.985	0.847	0.78
Zm00001d016072	50S ribosomal protein L21	NE plastid ribosome	1.085	0.243	NA	0.948	0.394	0.80
Zm00001d018096	50S ribosomal protein L29	NE plastid ribosome	0.964	0.746	NA	0.804	0.223	0.43
Zm00001d018412	50S ribosomal protein L9	NE plastid ribosome	1.089	0.007	0.82	0.974	0.674	0.75
Zm00001d019898	Plastid-specific 30S ribosomal protein 2	NE plastid ribosome	1.131	0.065	0.70	0.740	0.003	0.79
Zm00001d019898	Plastid-specific 30S ribosomal protein 2	NE plastid ribosome	1.125	0.014	0.70	0.740	0.003	0.79
Zm00001d025616	30S ribosomal protein 3	NE plastid ribosome	1.089	0.009	0.73	1.021	0.727	0.64
Zm00001d027421	50S ribosomal protein L11	NE plastid ribosome	1.117	0.015	0.73	0.820	0.026	0.66
Zm00001d027442	50S ribosomal protein L5	NE plastid ribosome	1.111	0.007	0.86	0.952	0.252	0.77
Zm00001d028153	30S ribosomal protein S10	NE plastid ribosome	1.044	0.279	NA	0.876	0.042	0.53
Zm00001d028240	50S ribosomal protein L15	NE plastid ribosome	1.108	0.101	0.82	0.833	0.002	0.54
Zm00001d028702	50S ribosomal protein L10	NE plastid ribosome	1.083	0.010	0.68	0.909	0.170	0.54
Zm00001d029201	50S ribosomal protein L6	NE plastid ribosome	1.132	0.016	0.84	0.886	0.026	0.41
Zm00001d032420	30S ribosomal protein S5	NE plastid ribosome	1.098	0.011	0.75	0.828	0.008	0.74
Zm00001d032896	50S ribosomal protein L28	NE plastid ribosome	1.038	0.436	0.67	0.827	0.026	0.79
Zm00001d033665	30S ribosomal protein S13	NE plastid ribosome	1.060	0.337	0.65	0.949	0.377	0.41
Zm00001d034192	30S ribosomal protein S9	NE plastid ribosome	1.103	0.039	0.70	0.826	0.010	0.49
Zm00001d034724	50S ribosomal protein L18	NE plastid ribosome	1.103	0.097	0.63	0.741	0.003	0.63
Zm00001d034808	30S ribosomal protein S6 alpha	NE plastid ribosome	1.134	0.043	0.75	0.756	0.009	0.79
Zm00001d034897	Plastid-specific 30S ribosomal protein 1	NE plastid ribosome	1.112	0.148	0.55	0.893	0.054	0.51
Zm00001d038084	50S ribosomal protein L1	NE plastid ribosome	1.118	0.010	0.71	0.909	0.028	0.78
Zm00001d041322	50S ribosomal protein L14	NE plastid ribosome	0.970	0.039	NA	0.873	0.030	0.29

Zm00001d043972	50S ribosomal protein L12-1	NE plastid ribosome	1.138	0.005	0.67	0.864	0.052	0.83
Zm00001d044130	50S ribosomal protein L31	NE plastid ribosome	1.136	0.071	0.77	1.039	0.396	0.51
Zm00001d046183	50S ribosomal protein L1	NE plastid ribosome	0.948	0.015	NA	1.064	0.831	-0.51
Zm00001d047462	50S ribosomal protein L6	NE plastid ribosome	1.097	0.009	0.79	0.886	0.154	0.72
Zm00001d047581	30S ribosomal protein S1	NE plastid ribosome	1.106	0.038	0.81	0.824	0.018	0.69
Zm00001d053377	50S ribosomal protein L21	NE plastid ribosome	1.061	0.462	NA	0.667	0.017	0.87
Zm00001d046835	rpo2	NE plastid RNA polymerase	NA	NA	NA	0.585	0.168	0.36
ZemaCp003.1	matK	PE other	1.022	0.488	NA	NA	NA	NA
ZemaCp024.1	ycf3	PE other	1.070	0.319	0.73	NA	NA	NA
ZemaCp035.1	cemA	PE other	1.211	0.013	NA	NA	NA	NA
ZemaCp047.1	clpP	PE other	1.060	0.147	0.75	NA	NA	NA
ZemaCp057.1	infA	PE other	1.092	0.021	0.18	NA	NA	NA
ZemaCp085.1	ccsA	PE other	1.043	0.089	0.59	NA	NA	NA
ZemaCp004.1	rps16	PE plastid ribosome	1.002	0.976	0.90	NA	NA	NA
ZemaCp016.1	rps2	PE plastid ribosome	1.087	0.010	0.90	NA	NA	NA
ZemaCp021.1	rps14	PE plastid ribosome	1.097	0.160	0.84	NA	NA	NA
ZemaCp026.1	rps4	PE plastid ribosome	1.108	0.003	0.83	NA	NA	NA
ZemaCp044.1	rpl33	PE plastid ribosome	1.024	0.072	0.65	NA	NA	NA
ZemaCp045.1	rps18	PE plastid ribosome	1.065	0.024	0.59	NA	NA	NA
ZemaCp046.1	rpl20	PE plastid ribosome	1.117	0.052	0.79	NA	NA	NA
ZemaCp055.1	rps11	PE plastid ribosome	0.997	0.979	0.52	NA	NA	NA
ZemaCp056.1	rpl36	PE plastid ribosome	0.950	0.606	NA	NA	NA	NA
ZemaCp058.1	rps8	PE plastid ribosome	1.091	0.007	0.81	NA	NA	NA
ZemaCp059.1	rpl14	PE plastid ribosome	1.056	0.033	0.40	NA	NA	NA
ZemaCp060.1	rpl16	PE plastid ribosome	1.083	0.012	0.67	NA	NA	NA
ZemaCp061.1	rps3	PE plastid ribosome	1.080	0.043	0.80	NA	NA	NA
ZemaCp062.1	rpl22	PE plastid ribosome	1.078	0.063	0.80	NA	NA	NA
ZemaCp063.1	rps19	PE plastid ribosome	1.122	0.017	0.80	NA	NA	NA
ZemaCp065.1	rpl2	PE plastid ribosome	1.080	0.001	0.76	NA	NA	NA
ZemaCp067.1	rpl23	PE plastid ribosome	1.114	0.074	0.78	NA	NA	NA
ZemaCp082.1	rps15	PE plastid ribosome	1.082	0.002	0.66	NA	NA	NA
ZemaCp084.1	rpl32	PE plastid ribosome	1.061	0.035	NA	NA	NA	NA
ZemaCp013.1	rpoB	PE plastid RNA polymerase	0.935	0.253	NA	NA	NA	NA

ZemaCp014.1	rpoC1	PE plastid RNA polymerase	0.897	0.064	NA	NA	NA	NA
ZemaCp054.1	rpoA	PE plastid RNA polymerase	0.843	0.023	0.62	NA	NA	NA
ZemaCp008.1	psbC	PhAPGs	1.483	0.000	0.22	NA	NA	NA
ZemaCp017.1	atpI	PhAPGs	1.378	0.024	0.07	NA	NA	NA
ZemaCp018.1	atpH	PhAPGs	1.311	0.222	NA	NA	NA	NA
ZemaCp019.1	atpF	PhAPGs	1.345	0.002	0.58	NA	NA	NA
ZemaCp022.1	psaB	PhAPGs	1.501	0.000	0.40	NA	NA	NA
ZemaCp023.1	psaA	PhAPGs	1.519	0.000	0.27	NA	NA	NA
ZemaCp027.1	ndhJ	PhAPGs	1.325	0.001	0.53	NA	NA	NA
ZemaCp028.1	ndhK	PhAPGs	1.313	0.012	0.31	NA	NA	NA
ZemaCp029.1	ndhC	PhAPGs	1.285	0.039	NA	NA	NA	NA
ZemaCp030.1	atpE	PhAPGs	1.250	0.004	0.68	NA	NA	NA
ZemaCp032.1	rbcL	PhAPGs	1.274	0.000	0.59	NA	NA	NA
ZemaCp036.1	petA	PhAPGs	1.551	0.000	0.63	NA	NA	NA
ZemaCp039.1	psbF	PhAPGs	1.462	0.000	0.08	NA	NA	NA
ZemaCp040.1	psbE	PhAPGs	1.474	0.001	0.05	NA	NA	NA
ZemaCp043.1	psaJ	PhAPGs	1.250	0.223	NA	NA	NA	NA
ZemaCp048.1	psbB	PhAPGs	1.495	0.000	0.21	NA	NA	NA
ZemaCp050.1	psbN	PhAPGs	0.946	0.117	NA	NA	NA	NA
ZemaCp051.1	psbH	PhAPGs	1.576	0.000	0.45	NA	NA	NA
ZemaCp052.1	petB	PhAPGs	1.489	0.007	0.21	NA	NA	NA
ZemaCp053.1	petD	PhAPGs	1.562	0.000	0.57	NA	NA	NA
ZemaCp075.1	ndhB	PhAPGs	1.336	0.000	0.45	NA	NA	NA
ZemaCp083.1	ndhF	PhAPGs	1.328	0.002	0.36	NA	NA	NA
ZemaCp086.1	ndhD	PhAPGs	1.337	0.003	0.42	NA	NA	NA
ZemaCp087.1	psaC	PhAPGs	1.482	0.000	0.36	NA	NA	NA
ZemaCp088.1	ndhE	PhAPGs	1.322	0.008	NA	NA	NA	NA
ZemaCp090.1	ndhI	PhAPGs	1.294	0.005	0.37	NA	NA	NA
ZemaCp091.1	ndhA	PhAPGs	1.354	0.003	0.26	NA	NA	NA
ZemaCp092.1	ndhH	PhAPGs	1.330	0.001	0.54	NA	NA	NA
Zm00001d009723	plastid transcriptionally active14	PTAC	0.937	0.058	0.73	0.840	0.036	0.45
Zm00001d009877	Protein plastid transcriptionally active 16	PTAC	1.256	0.000	-0.11	0.846	0.003	0.64
Zm00001d018401	plastid transcriptionally active 17	PTAC	1.043	0.021	-0.29	1.175	0.076	0.55
Zm00001d018401	plastid transcriptionally active 17	PTAC	1.043	0.021	-0.34	1.175	0.076	0.55

Zm00001d018401	plastid transcriptionally active 17	PTAC	0.970	0.045	-0.29	1.175	0.076	0.55
Zm00001d018401	plastid transcriptionally active 17	PTAC	0.970	0.045	-0.34	1.175	0.076	0.55
Zm00001d022599	plastid transcriptionally active chromosome 2 homolog	PTAC	0.937	0.051	0.80	0.961	0.471	0.48
Zm00001d025848	plastid transcriptionally active 5	PTAC	1.117	0.034	-0.55	0.941	0.086	0.56
Zm00001d030153	plastid transcriptionally active 18	PTAC	0.971	0.484	NA	0.596	0.001	0.55
Zm00001d032790	plastid transcriptionally active 3	PTAC	0.957	0.147	0.80	1.000	0.998	0.18
Zm00001d032790	plastid transcriptionally active 3	PTAC	0.985	0.751	0.80	1.000	0.998	0.18
Zm00001d043325	plastid transcriptionally active chromosome 12 homolog	PTAC	0.899	0.030	0.55	0.786	0.023	0.57
Zm00001d052329	plastid transcriptionally active 6	PTAC	0.889	0.008	0.52	0.800	0.008	0.91
Zm00001d012810	sig6	Sigma factor	NA	NA	NA	1.054	0.298	0.63
Zm00001d024507	sig1A	Sigma factor	NA	NA	NA	1.137	0.032	0.66
Zm00001d028585	sig2B	Sigma factor	NA	NA	NA	1.109	0.092	0.69
Zm00001d039194	sig8	Sigma factor	1.242	0.019	NA	1.248	0.038	0.05
Zm00001d049160	sig2A	Sigma factor	NA	NA	NA	0.828	0.004	0.78
Zm00001d049494	sig1B	Sigma factor	NA	NA	NA	1.270	0.003	0.64

Supplemental Table 1. Protein and RNA Pearson's correlations and expression heterosis values with corresponding p-values for all plastid-encoded genes, their nuclear-encoded binding partners, and sigma factors.

Dataset S1 (separate file). Hybrid/mid-parent and parent/parent ratios for protein expression from the B73xMo17 hybrid and its parents.

Dataset S2 (separate file). Z-test scores for protein expression and correlations of the groups shown in Figures 1, 3, and 4.

Dataset S3 (separate file). Chromosomal breakpoints of the RILs and their hybrids. a=B73, b=Mo17, h=heterozygous.

Dataset S4 (separate file). Average plant heights of the RILs, diverse inbreds, and each of their hybrids used for correlation analyses.

Dataset S5 (separate file). Pearson correlations between expression heterosis (hybrid/mid-parent) of each protein and transcript and plant height heterosis (hybrid/mid-parent plant height).

Dataset S6 (separate file). Hybrid/mid-parent ratios and their p-values for protein and RNA expression of ethylene biosynthetic enzymes in the seedling leaf of the B73xMo17 hybrid.

Dataset S7 (separate file). Counts per million (CPM) values from RNA-seq analysis of seedling leaf tissue for three biological replicates each of six maize hybrids and their inbred parents.

Dataset S8 (separate file). Counts per million (CPM) values from RNA-seq analysis of seedling leaf tissue for three to four biological replicates each of eight maize RIL hybrids and their RIL parents, as well as the B73xMo17 hybrid and its parents.

Dataset S9 (separate file). Normalized tandem-mass tag (TMT) values for all experiments in this study.

Dataset S10 (separate file). Protein group assignments and their sources.

SI References

1. P. A. Ewels, *et al.*, The nf-core framework for community-curated bioinformatics pipelines. *Nat. Biotechnol.* **38**, 276–278 (2020).
2. Y. Jiao, *et al.*, Improved maize reference genome with single-molecule technologies. *Nature* **546**, 524–527 (2017).
3. D. Kim, B. Langmead, S. L. Salzberg, HISAT: A fast spliced aligner with low memory requirements. *Nat. Methods* **12**, 357–360 (2015).
4. Y. Liao, G. K. Smyth, W. Shi, FeatureCounts: An efficient general purpose program for assigning sequence reads to genomic features. *Bioinformatics* **30**, 923–930 (2014).
5. M. D. Robinson, A. Oshlack, A scaling normalization method for differential expression analysis of RNA-seq data. *Genome Biol.* **11** (2010).
6. S. Sun, *et al.*, Extensive intraspecific gene order and gene structural variations between Mo17 and other maize genomes. *Nat. Genet.* **50**, 1289–1295 (2018).
7. P. Zhou, C. N. Hirsch, S. P. Briggs, N. M. Springer, Dynamic Patterns of Gene Expression Additivity and Regulatory Variation throughout Maize Development. *Mol. Plant* **12**, 410–425 (2019).
8. H. Wickham, R. François, L. Henry, K. Müller, dplyr: A Grammar of Data Manipulation (2019).
9. H. Wickham, The Split-Apply-Combine strategy for data analysis. *J. Stat. Softw.* **40**, 1–29 (2011).
10. H. Wickham, L. Henry, tidyr: Easily Tidy Data with "spread()" and "gather()" Functions (2019).
11. H. Wickham, Reshaping Data with the {reshape} Package. *J. Stat. Softw.* **21**, 1–20 (2007).

12. H. Wickham, stringr: Simple, Consistent Wrappers for Common String Operations (2019).
13. M. Dowle, A. Srinivasan, data.table: Extension of `data.frame` (2019).
14. D. W. Huang, B. T. Sherman, R. A. Lempicki, Systematic and integrative analysis of large gene lists using DAVID bioinformatics resources. *Nat. Protoc.* **4**, 44–57 (2009).
15. D. W. Huang, B. T. Sherman, R. A. Lempicki, Bioinformatics enrichment tools: paths toward the comprehensive functional analysis of large gene lists. *Nucleic Acids Res.* **37**, 1–13 (2009).
16. H. Wickham, *ggplot2: Elegant Graphics for Data Analysis* (Springer-Verlag New York, 2016).
17. C. O. Wilke, cowplot: Streamlined Plot Theme and Plot Annotations for "ggplot2" (2019).
18. T. Pedersen, patchwork: The Composer of Plots (2019).
19. W. Luo, C. Brouwer, Pathview: an R/Bioconductor package for pathway-based data integration and visualization. *Bioinformatics* **29**, 1830–1831 (2013).
20. C. O. Wilke, ggridges: Ridgeline Plots in "ggplot2" (2020).
21. A. Arnholt, B. Evans, BSDA: Basic Statistics and Data Analysis (2017).
22. G. Friso, W. Majeran, M. Huang, Q. Sun, K. J. van Wijk, Reconstruction of metabolic pathways, protein expression, and homeostasis machineries across maize bundle sheath and mesophyll chloroplasts: Large-scale quantitative proteomics using the first maize genome assembly. *Plant Physiol.* **152**, 1219–1250 (2010).
23. M. Kanehisa, Toward understanding the origin and evolution of cellular organisms. *Protein Sci.* **28**, 1947–1951 (2019).
24. J. L. Portwood, *et al.*, Maizegdb 2018: The maize multi-genome genetics and genomics database. *Nucleic Acids Res.* **47**, D1146–D1154 (2019).
25. , National Center for Biotechnology Information (NCBI). *Natl. Libr. Med. (US), Natl. Cent. Biotechnol. Inf.* (1988).
26. K. Wimalanathan, I. Friedberg, C. M. Andorf, C. J. Lawrence-Dill, Maize GO Annotation—Methods, Evaluation, and Review (maize-GAMER). *Plant Direct* **2**, 1–15 (2018).
27. H. Pagès, M. Carlson, S. Falcon, N. Li, AnnotationDbi: Manipulation of SQLite-based annotations in Bioconductor (2019).
